# miR-34 modulates wing polyphenism in planthopper

**DOI:** 10.1371/journal.pgen.1008235

**Published:** 2019-06-26

**Authors:** Xinhai Ye, Le Xu, Xiang Li, Kang He, Hongxia Hua, Zhenghong Cao, Jiadan Xu, Wanyi Ye, Jiao Zhang, Zhuting Yuan, Fei Li

**Affiliations:** 1 State Key Laboratory of Rice Biology & Ministry of Agricultural and Rural Affairs Key Laboratory of Molecular Biology of Crop Pathogens and Insects, Institute of Insect Sciences, Zhejiang University, Hangzhou, China; 2 Hubei Insect Resources Utilization and Sustainable Pest Management Key Laboratory, College of Plant Science and Technology, Huazhong Agricultural University, Wuhan, China; 3 Department of Entomology, Nanjing Agricultural University, Nanjing, China; New York University, UNITED STATES

## Abstract

Polyphenism is a successful strategy adopted by organisms to adapt to environmental changes. Brown planthoppers (BPH, *Nilaparvata lugens*) develop two wing phenotypes, including long-winged (LW) and short-winged (SW) morphs. Though insulin receptor (*InR*) and juvenile hormone (JH) have been known to regulate wing polyphenism in BPH, the interaction between these regulators remains largely elusive. Here, we discovered that a conserved microRNA, *miR-34*, modulates a positive autoregulatory feedback loop of JH and insulin/IGF signaling (IIS) pathway to control wing polyphenism in BPH. *Nlu-miR-34* is abundant in SW BPHs and suppresses *NlInR1* by targeting at two binding sites in the 3’UTR of *NlInR1*. Overexpressing *miR-34* in LW BPHs by injecting agomir-34 induces the development towards SW BPHs, whereas knocking down *miR-34* in SW BPHs by injecting antagomir-34 induces more LW BPHs when another *NlInR1* suppressor, *NlInR2*, is also suppressed simultaneously. A cis-response element of Broad Complex (*Br-C*) is found in the promoter region of *Nlu-miR-34*, suggesting that 20-hydroxyecdysone (20E) might be involved in wing polyphenism regulation. Topic application of 20E downregulates *miR-34* expression but does not change wing morphs. On the other hand, JH application upregulates *miR-34* expression and induces more SW BPHs. Moreover, knocking down genes in IIS pathway changes JH titers and *miR-34* abundance. In all, we showed that miRNA mediates the cross talk between JH, 20E and IIS pathway by forming a positive feedback loop, uncovering a comprehensive regulation mechanism which integrates almost all known regulators controlling wing polyphenism in insects.

## Author summary

Polyphenism is a fascinating phenomenon which significantly improves the ability of a species to explore various environmental resources. Brown planthopper (*Nilaparvata lugens*, BPH) is a notorious insect pest which causes huge damages to rice. BPH has two wing phenotypes, long-winged (LW) and short-winged (SW) morphologies. LW morphs are capable of long-distance migration, while SW morphs have high reproductive capabilities. Juvenile hormone (JH) and insulin/IGF signaling (IIS) pathway have been known to participate in regulating polyphenism in various organisms. However, how these regulators interact with each other remains largely unknown. Here, we show that a conserved microRNA, *miR-34*, mediates the crosstalk between JH, 20E and IIS pathway to modulate wing polyphenism in BPH. *miR-34* suppresses insulin receptor-1(*InR1*), increases JH titer, and induces SW morphs. On the other hand, JH increases the expression of *miR-34* and induces SW morphs, while 20E decreases *miR-34* but does not change proportion of wing morphs. Knocking down genes in IIS pathway changes JH titer and *miR-34* abundance. Therefore, miRNA, JH, 20E and IIS form an autoregulatory feedback loop to control wing polyphenism in BPH. Our work presents a comprehensive mechanism of wing polyphenism by integrating various regulators.

## Introduction

The phenomenon of polyphenism is that two or more distinct phenotypes are displayed by an organism with the same genotype. This phenomenon is triggered by environmental cues such as population density, host nutrition, and temperature [1]. For example, locusts show density-dependent phenotypic plasticity and have two phases: a low-density “solitarious” phenotype and a high-density “gregarious” phenotype [2]. In beetles, horn polyphenism is a nutrition-dependent trait where some male beetles have fully-developed horns, while other males are completely hornless, depending on their nutrition status and body size [3]. Eusocial insects, including members of Hymenoptera, Blattodea (termites), often display caste differentiation, producing multiple types of offspring with different reproductive and morphological features [4]. Based on the physiological state of the mother, aphids produce winged adults in deteriorating environments and flightless morphs when environmental conditions are stable [5].

Brown planthopper (BPH, *Nilaparvata lugens*) is one of the most notorious planthoppers, migrating from Vietnam and the Philippines to China and Japan in summer during the rice growing season, then flying back to tropical regions during winter after rice crops have been harvested in temperate zones [6]. Wing polyphenism is a key determinant of the success of planthoppers [7]. Long-winged (LW) BPHs are capable of long-distance migration, while short-winged (SW) morphs display higher reproductive capabilities. Prior works showed that insulin/IGF-1 signaling (IIS) pathways involved in regulating wing polyphenism in BPHs [8]. Two insulin receptors (*InR*), *NlInR1* and *NlInR2*, have been shown to play opposing roles in determining wing fate. *NlInR2* inhibits *NlInR1*, switching long-winged morphs to short-winged morphs, and vice versa. These two insulin receptors modulate wing polyphenism by regulating insulin/IGF signaling (IIS) pathway [9]. Wounding causes a shift to short wing through stimulation of the forkhead transcription factor (*NlFoxO*) and its downstream target *Nl4EBP* [10]. High glucose concentration in the rice host induces long-winged females, indicating that host nutrition quality has a direct impact on wing polyphenism in female BPH. This work shows that the nutrition is a key determination factor in controlling wing polyphenism in BPH and also raises a question of sex difference in wing polyphenism [11]. Silencing the c-Jun NH2-terminal kinase (*NlJNK*) increased the proportion of short winged female adults, suggesting that JNK signaling involve in regulating wing polyphenism in BPH [12]. In addition, topic application of juvenile hormone (JH) in the BPH nymphs induces SW morphs [13]. Knocking down the juvenile hormone epoxide hydrolase (*Nljheh*) prevents the degradation of JH and induces a bias towards SW BPHs [14], providing evidence that JH might also involve in regulating wing polyphenism. However, there is a dispute over JH regulation of wing polyphenism in BPH [8, 15].

Though various regulators have been reported to involve in modulating phenotypic plasticity of wing polyphenism in BPHs, how these regulators interact with each other remains an important unsolved question. miR-34 is an important modulator involved in the cross talk between the IIS pathway and hormone in *C*. *elegans* and *Drosophila*. In *C*. *elegans*, *miR-34* expression is regulated by IIS via a negative feedback loop between *miR-34* and *DAF-16* (the sole ortholog of *FoxO* in nematodes), providing robustness to environmental stress [16]. *miR-34* is maternally inherited in *Drosophila* [17], and is activated by JH but is suppressed by 20E through Broad Complex (*Br-C*) [18]. Here, we found a microRNA (miRNA), *Nlu-miR-34*, modulates the crosstalk between IIS, JH and 20-hydroxyecdysone (20E) pathways to control wing polyphenism. *Nlu-miR-34* suppresses *NlInR1*, inducing the development towards SW morph. Further, *Nlu-miR-34* is activated by JH through *Br-C*. Moreover, silencing *NlInR1* induces the expression of *Nlu-miR-34*, suggesting that *Nlu-miR-34*, IIS, and JH form an autoregulatory positive feedback loop to control wing polyphenism in BPHs.

## Results

### *Nlu-miR-34* controls *NlInR1* in BPHs

**Fig 1:**
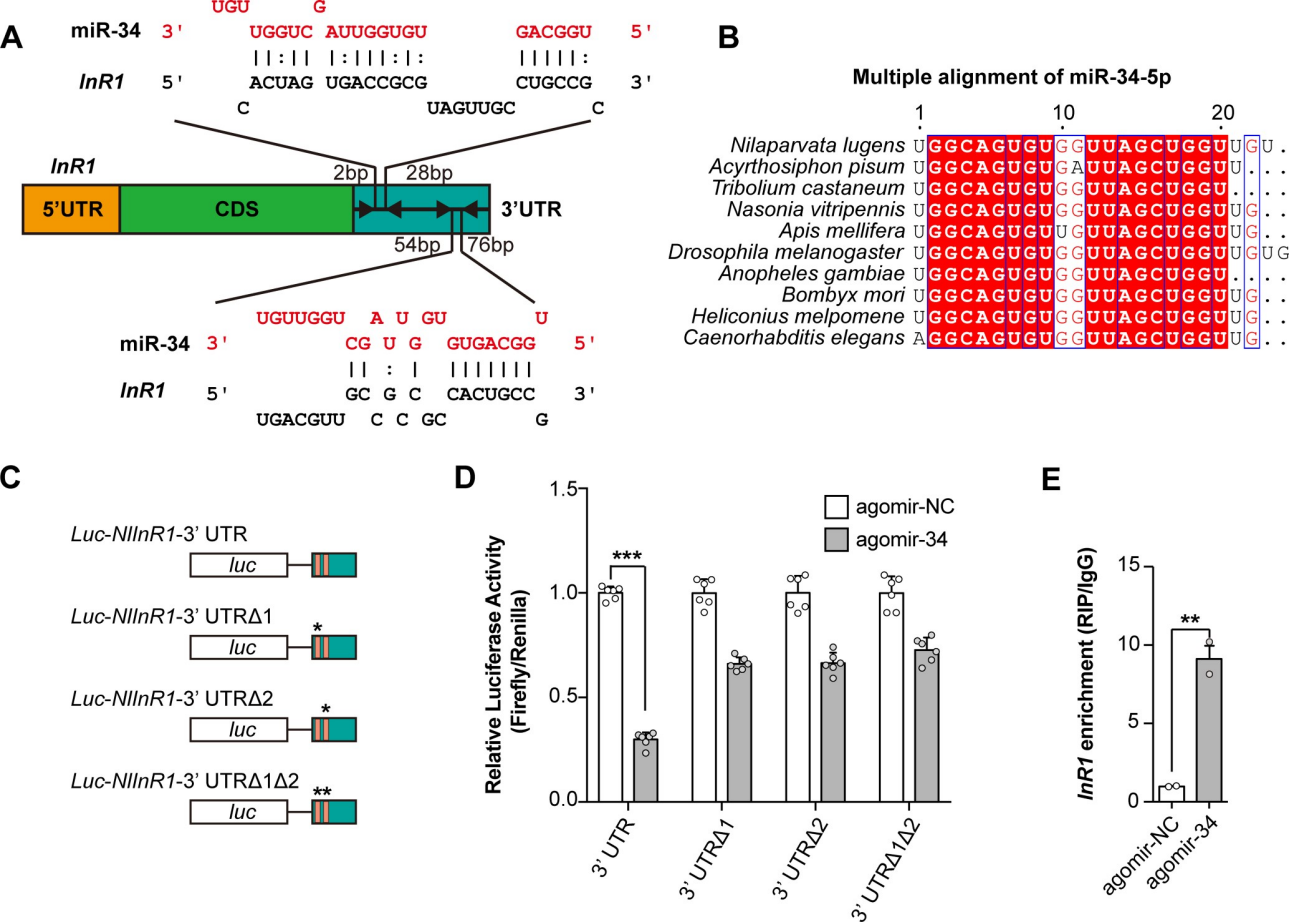
*Nlu-miR-34* targets insulin receptor-1 (*NlInR1*). (A) Two binding sites were predicted in the 3’UTR of *NlInR1* by miRanda, TargetScan, microTar, PITA, and RNAhybrid. (B) Multiple alignment of miR-34-5p in insects and *Caenorhabditis elegans*. (C) Schematic mutation of two predicted *Nlu-miR-34* binding sites within the *Luc-NlInR1-3’UTR* luciferase reporter. Asterisks means the mutated miRNA binding sites. (D) Dual luciferase reporter assays confirmed the interactions between *Nlu-miR-34* and *NlInR1 in vitro*. The relative luciferase activities were significantly decreased in the presence of agomir-34. Mutating either of the binding sites abolished the repression effect of agomir-34. Data are means ± SEM, *p* = 4.99e^-12^, six replicates. (E) RNA-binding protein immunoprecipitation (RIP) confirmed the interactions between *Nlu-miR-34* and *NlInR1 in vivo*. Transcripts of *NlInR1* were significantly enriched by antibody against Ago1 in agomir-34 treated group compared with it in agomir-NC treated group. Data are means ± SEM, *p* = 0.002, n = 50, two replicates. **p < 0.01, ***p < 0.001 (Student’s *t*-test).

Insulin receptors in BPH (*NlInRs*) were reported to determine the wing polyphenism in BPH [9]. To investigate whether miRNA may play a role in regulating wing polyphenism, we carried out a bioinformatics analysis to identify miRNAs targeting at *NlInR1* or *NlInR2*. The results showed that only *Nlu-miR-34* can target *NlInR1* with two putative binding sites in the 3^’^untranslated region (UTR) predicted by all five target prediction algorithms (**Fig 1A**, see methods). *miR-34* is highly conserved in insects and nematodes (**Fig 1B**, see methods) and is predicted to have 19 target genes in BPH (**S1 Table**). To confirm the interaction between *Nlu-miR-34* and *NlInR1*, we introduced the 3’UTR sequence of *NlInR1* at the downstream of the firefly luciferase gene in the pMIR-REPORT vector. Constructs with or without (negative control) the 3’UTR of *NlInR1* were both transfected into human embryonic kidney 293T (HEK293T) cells. The luciferase activity was significantly reduced to 30% relative to the negative control in the presence of agomir-34 (the mimics of *Nlu-miR-34*) (**Fig 1C and 1D**, Student’s *t*-test, *p* = 4.99e^-12^, n = 300, six replicates). Mutating any of the binding sites abolished the suppression effect of agomir-34 on the reporter activity of the *NlInR1* target sites (**Fig 1D**), indicating that both two binding sites are essential for the interaction between *Nlu-miR-34* and *NlInR1*. Next, we performed RNA-binding protein immunoprecipitation (RIP) assay with the antibody-Ago1 (Argonaute 1) in wing buds of BPH nymphs. The transcripts of *NlInR1* was significantly enriched in the Ago1-immunoprecipitated RNAs from agomir-34-injected BPHs compared with those from the control samples (**Fig 1E**, 9.30-fold, Student’s *t*-test, *p* = 0.002, n = 150, two replicates), showing that *Nlu-miR-34* directly interacted with *NlInR1* in the wing buds *in vivo*. Then, we used fluorescence *in situ* hybridization (FISH) assay to examine whether *Nlu-miR-34* co-localize with *NlInR1* in wing buds. Indeed, both of *Nlu-miR-34* and *NlInR1* were detected in wing buds of the fourth instar BPHs (**S1 Fig**).

### *Nlu-miR-34* is highly expressed in SW nymphs in critical stages of wing fate determination

**Fig 2:**
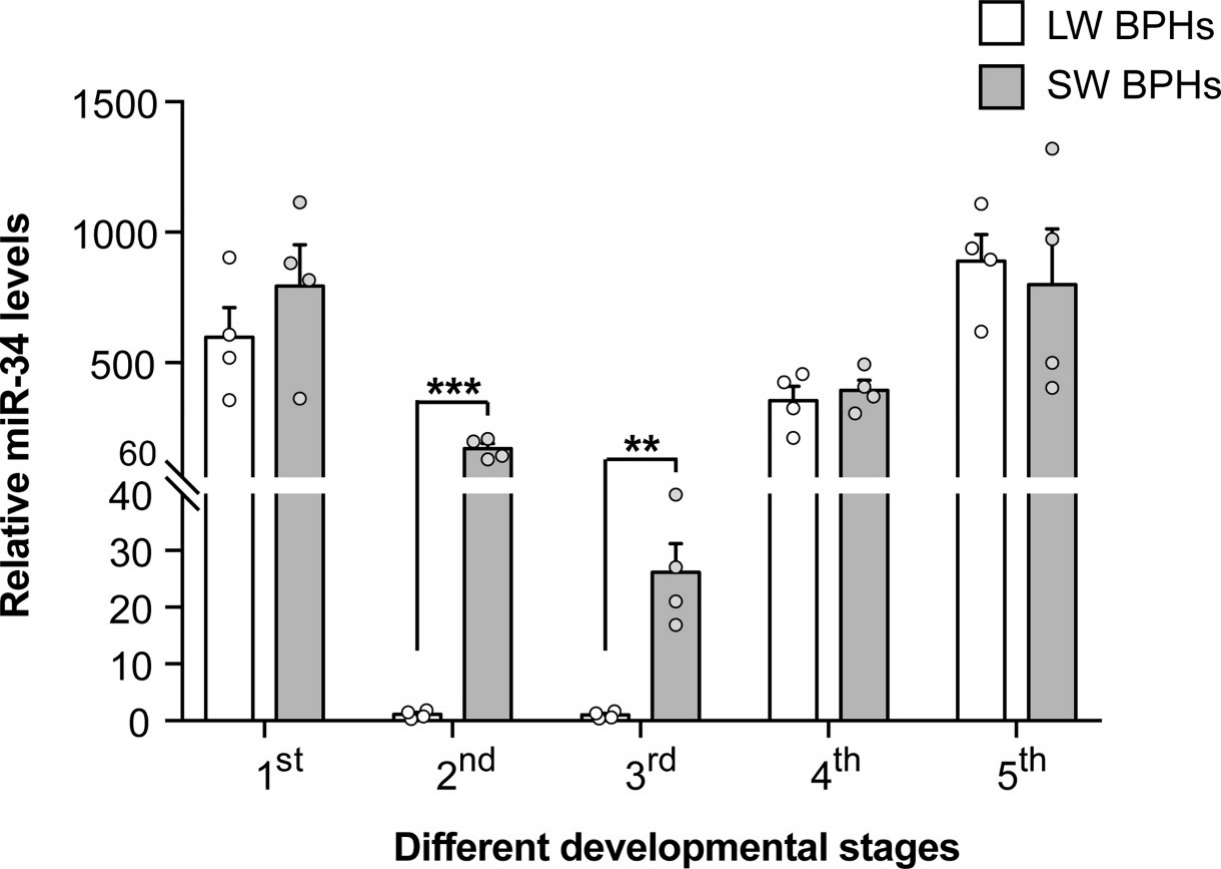
*Nlu-miR-34* is highly expressed in SW nymphs in critical stages of wing fate determination. *Nlu-miR-34* is highly abundant in the second and third instar SW strain nymphs, indicating that *Nlu-miR-34* is expressed during critical stages of wing fate determination. The qPCR data are presented as means ± SEM, *p* = 0.0001 for the second instar, and *p* = 0.002 for the third instar, four replicates. **p < 0.01, ***p < 0.001 (Student’s *t*-test).

The critical stages of wing fate determination in BPH is the second to fourth instar of nymph [19]. However, the wing morphs cannot be discriminated at nymph stages. To examine the expression of *Nlu-miR-34* in nymphs with different wing fate, we first developed two BPH strains by continually selecting SW or LW adults for crossing for more than 50 generations (SW♀ x SW♂ for SW strain, and LW♀ x LW♂ for LW strain) [14]. Almost all adults in SW strain emerge as short-winged adults while 80% of LW strain individuals become long-winged adult morphs. Because these two strains were selected from the same population, SW and LW strain should have the same genetic background. Each generation was purified in the adult stage using a previously described method [20]. We measured the expression of *Nlu-miR-34* in the nymph stages of both SW and LW strain. The results show that *Nlu-miR-34* was significantly highly expressed in the second and third instars nymphs in SW strain (**Fig 2,** Student’s *t*-test, *p* = 0.0001 for the second instar, and *p* = 0.002 for the third instar, four replicates), suggesting that *Nlu-miR-34* might be essential to develop SW adults. However, we found that *NlInR1* was also highly expressed in the second and third instar SW nymphs when using the whole body for experiments (**S2 Fig**).

### Overexpressing *Nlu-miR-34* induces SW morphs by repressing *NlInR1*

**Fig 3:**
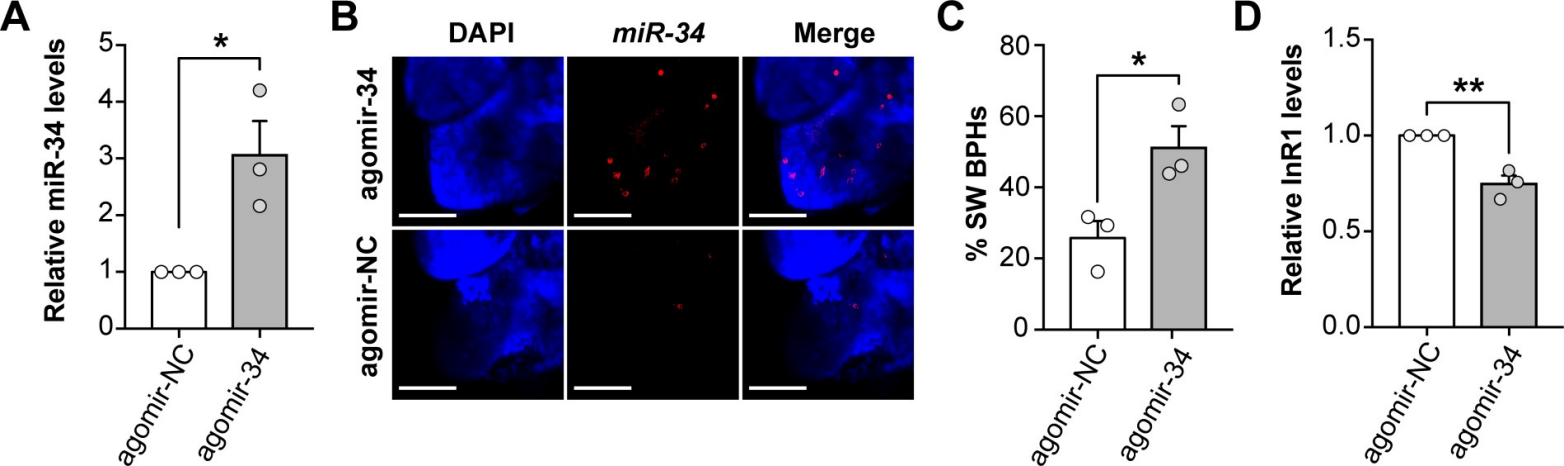
Overexpression of *Nlu-miR-34* in LW strain induced the development towards SW BPHs by suppressing *NlInR1*. (A) The agomir-34 was injected into early third instar nymphs in LW strain, significantly increasing the *Nlu-miR-34* expression (*p* = 0.026). (B) Fluorescence in situ hybridization assay (FISH) showed that *Nlu-miR-34* was more abundant in wing buds treated with agomir-34, relative to the negative control (agomir-NC). Scale bars:100 μm. (C) Injection of nymphs with agomir-34 induced a strong bias towards SW BPHs compared to the control (*p* = 0.032, n = 150). (D) The relative abundance of *NlInR1* (*p* = 0.004) was significantly decreased after nymphs were injected with agomir-34. The qPCR and wing rate data are presented as means ± SEM, three replicates. *p < 0.05, **p < 0.01 (Student’s *t*-test).

To decipher the roles of *Nlu-miR-34* in controlling wing polyphenism, we increased its abundance by injecting agomir-34 into the third instar nymphs at 24 h post molt in LW strain. The abundance of *Nlu-miR-34* was significantly increased by 3.06-fold compared to the negative control, which was treated with agomir-NC (a synthesized small RNA with a randomly shuffled sequence of *Nlu-miR-34*) (**Fig 3A**, Student’s *t*-test, *p* = 0.026, three replicates). Fluorescence in situ hybridization (FISH) assay showed that the abundance of *Nlu-miR-34* increased in the wing bud cells at 24 h post injection (**Fig 3B**). Next, the agomir-34 and agomir-NC treated nymphs were kept for wing observation until emergence into adults. The results showed that overexpression of *Nlu-miR-34* induced the development of SW morphs 51.08 ± 6.16% in a significantly higher proportion than those in the control group 25.78 ± 4.78% (**Fig 3C**, Student’s *t*-test, *p* = 0.032, n = 150, three replicates). In addition, overexpression of *Nlu-miR-34* downregulated the expression of its target gene *NlInR1*(**Fig 3D**, Student’s *t*-test, *p* = 0.005, three replicates).

### *Nlu-miR-34* is a supplemental suppressor on *NlInR1*

**Fig 4:**
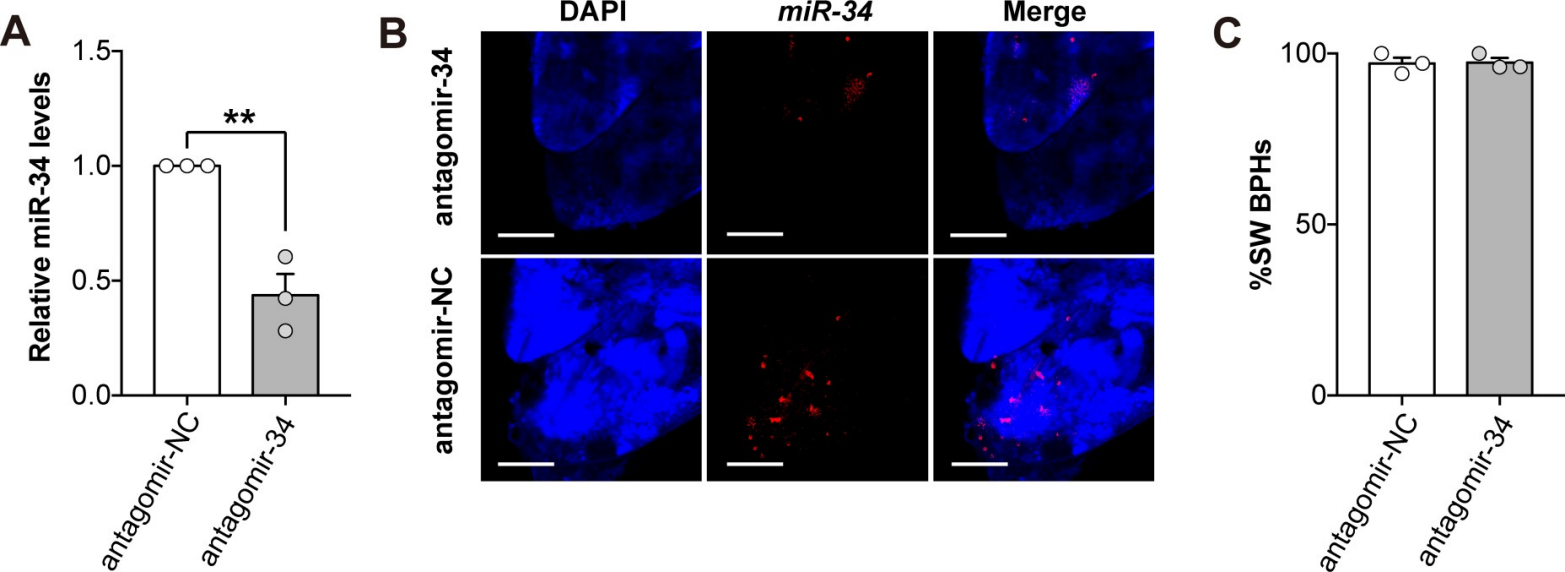
Inhibition of *Nlu-miR-34* in SW strain by antagomir-34. (A) The expression of *Nlu-miR-34* was significantly decreased by injecting antagomir-34 (*p* = 0.004). (B) Fluorescence in situ hybridization assay (FISH) showed that *Nlu-miR-34* was less abundant in the wing bud treated with antagomir-34, relative to the negative control (antagomir-NC). Scale bars:100 μm. (C) Inhibition of *Nlu-miR-34* by antagomir-34 does not induce changes in wing morphology (n = 50). The qPCR and wing rate data are presented as means ± SEM, three replicates. **p < 0.01 (Student’s *t*-test).

**Fig 5:**
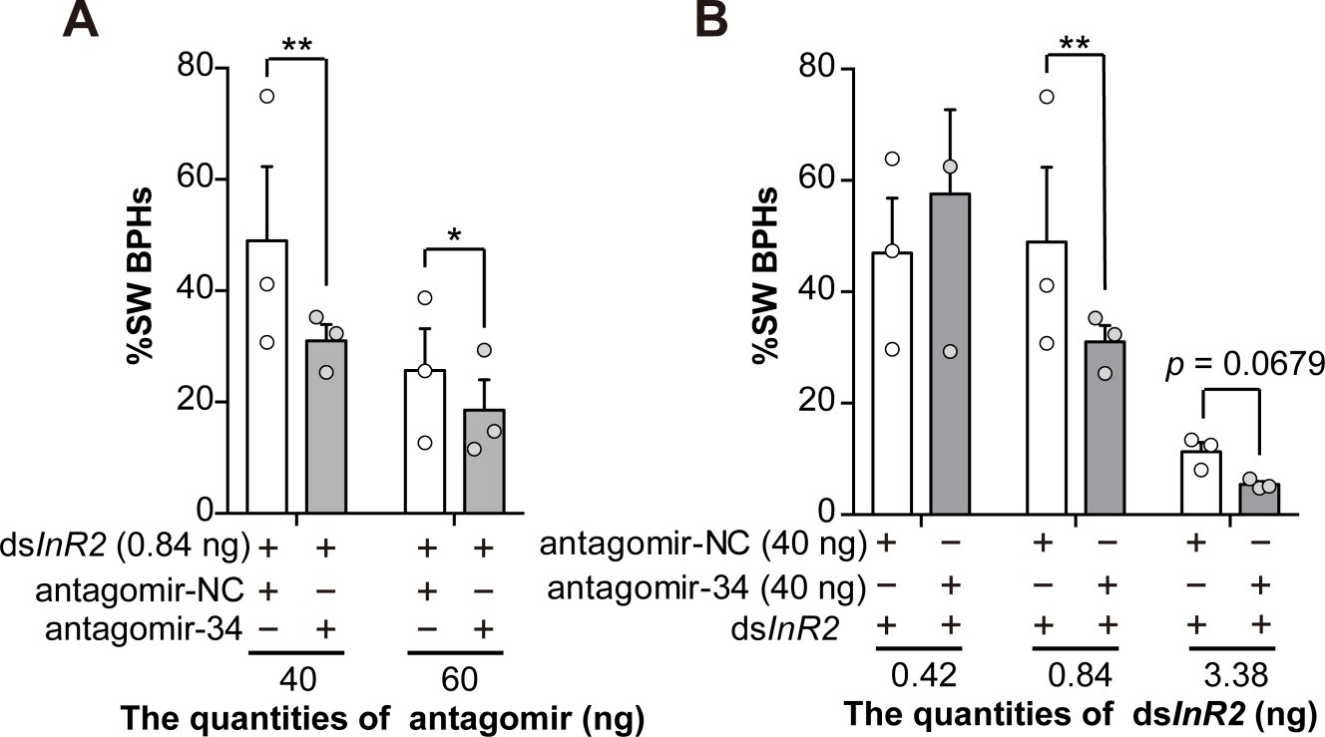
Simultaneously inhibition *Nlu-miR-34* and *NlInR2* in SW strain induced the decrease of SW BPHs. (A) Antagomir-34 (with two different quantities) and ds*NlInR2* (0.84 ng) were injected into the third instar SW strain nymphs, showed that injection of antagomir-34 decreased the ratio of SW type BPH (Chi-square test, 40ng: χ^2^ = 5.8, df = 1, *p* = 0.003; 60ng: χ^2^ = 7.9, df = 1, *p* = 0.042). (B) The antagomir-34 (40 ng) and ds*NlInR2* (with three different quantities) were simultaneously injected into the third instar SW strain nymphs. The results showed that effect of low level of ds*NlInR2* (0.42 ng) with antagomir-34 is familiar with control, while higher level of ds*NlInR2* (0.84 and 3.38 ng) with antagomir-34 induced a bias towards the development of LW BPHs compared to the control. Injection of antagomir-34 (40 ng) and ds*NlInR2* (0.84 ng) decreased ratio of SW BPH to around 20% (Chi-square test, χ^2^ = 8.4, df = 1, *p* = 0.004). High level of ds*InR2* (3.38 ng) with antagomir-34 decreased the ratio of SW BPH (Chi-square test, χ^2^ = 3.3, df = 1, *p* = 0.068). 200 insects were used for each experiment. Data are means ± SEM, three replicates. *p < 0.05, **p < 0.01.

Having showed increased *Nlu-miR-34* in LW strain induces SW morphs, we next inhibited *Nlu-miR-34* in SW strain by injecting antagomir-34 (the inhibitor of *Nlu-miR-34*). The expression of *Nlu-miR-34* was significantly decreased by 56.33 ± 16.16% (**Fig 4A**, Student’s *t*-test, *p* = 0.004, three replicates). FISH assay showed that the abundance of *Nlu-miR-34* decreased in the wing bud cells at 24 h post injection (**Fig 4B**). However, we did not observe a decrease of SW morphs in antagomir-34 treated groups comparing with the negative control (**Fig 4C**, Student’s *t*-test, *p* = 0.897, n = 150, three replicates). Since *NlInR2* suppresses *NlInR1* by forming a heterodimer [9] and *NlInR2* is highly expressed in SW strain (**S3 Fig**), we speculated that *NlInR2* might have a more potent suppressing effect and thus impair the effect of antagomir-34. To test this hypothesis, we first knocked down *NlInR2* with 0.84 ng ds*NlInR2* and then inhibited *Nlu-miR-34* with two different amounts of antagomir-34 (40 ng and 60 ng) in SW strain. As expected, after partially knocking down *NlInR2* by injecting ds*NlInR2*, downregulation of *Nlu-miR-34* significantly induced LW morphs (**Fig 5A**, Chi-square test, 40ng: χ^2^ = 5.8, df = 1, *p* = 0.003; 60ng: χ^2^ = 7.9, df = 1, *p* = 0.042; n = 150, three replicates). To test whether this suppressing effect is quantity dependent, we next inhibit *NlInR2* by injecting three different amounts of ds*NlInR2* (0.42 ng, 0.84 ng and 3.38 ng). The ds*NlInR2*-treated individuals were further injected with 40 ng antagomir-34 to inhibit *Nlu-miR-34*. The results showed that at a low quantity of ds*NlInR2* (0.42 ng), knocking down *Nlu-miR-34* with 40 ng antagomir-34 did not lead to more LW morphs (**Fig 5B**). However, when ds*NlInR2* increased to 0.84 ng and 3.38 ng, the proportions of LW BPHs was significantly increased and SW BPHs were significantly deceased (**Fig 5B**, Chi-square test, 0.84 ng: χ^2^ = 8.4, df = 1, *p* = 0.004; 3.38 ng: χ^2^ = 3.3, df = 1, *p* = 0.068; n = 150, three replicates). These results suggested that both *NlInR2* and *Nlu-miR-34* suppress *NlInR1* to control wing polyphenism. *NlInR2* might have a major suppression effect whereas *Nlu-miR-34* might be a “supplemental” suppressor to clear the leaky transcripts of *NlInR1* in the wing bud of SW strain.

### *Nlu-miR-34* is upregulated by JH through *Br-C*

**Fig 6:**
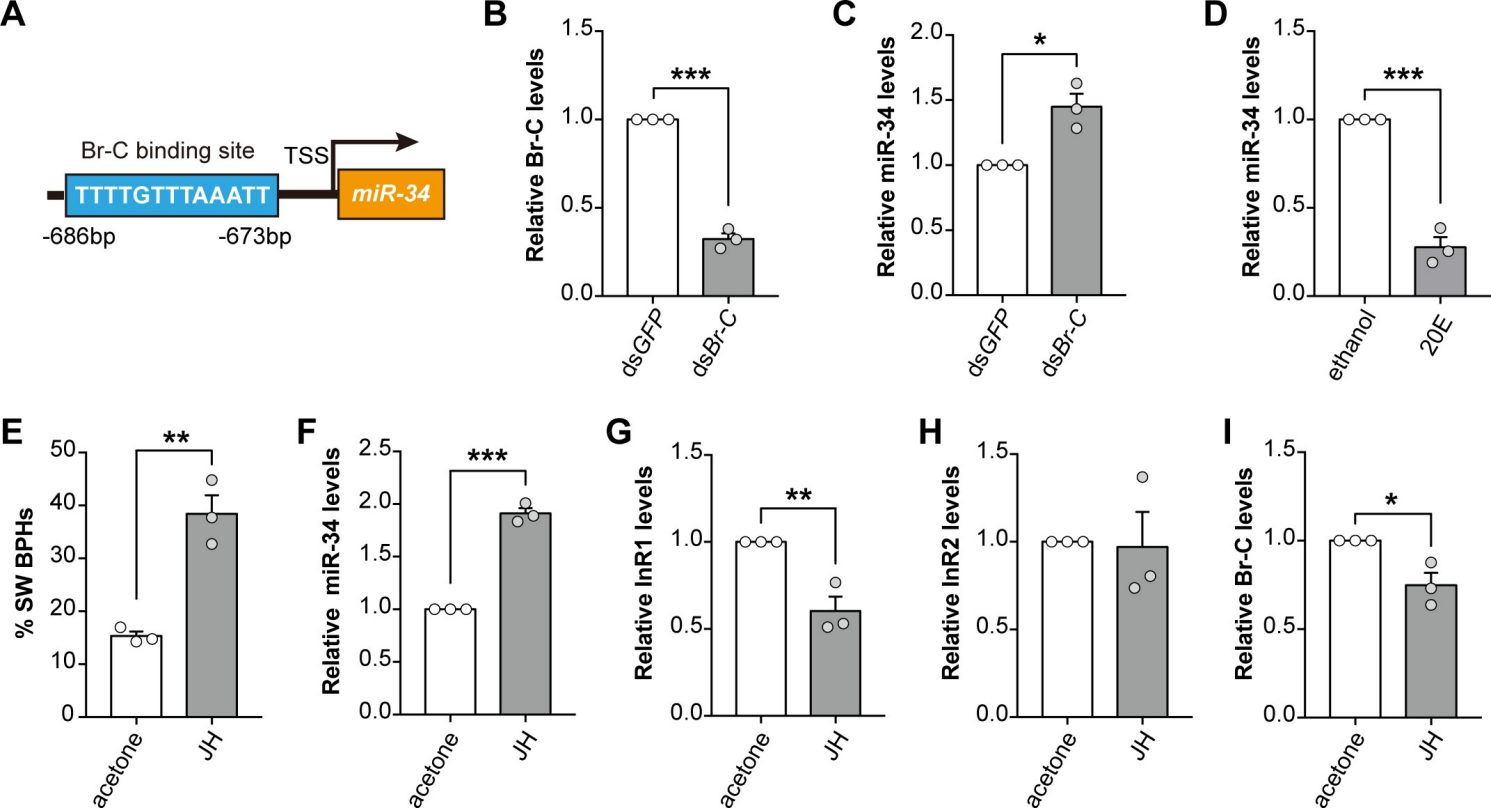
*Nlu-miR-34* is upregulated by JH through *Br-C*. (A) A cis-response element specific to *NlBr-C* Z4 activity was identified in the promoter region of *Nlu-miR-34*. (B) Expression of *NlBr-C* was successfully knocked down in LW strain after treating with ds*NlBr-C* (*p* = 3.21e^-5^). (C) Knockdown of *NlBr-C* leads to the increase of *Nlu-miR-34* transcripts (*p* = 0.011). (D) 20E application in SW strain results in the decreased of *Nlu-miR-34* (*p* = 0.0001, four replicates). (E) Application of JH in LW-strain induced a strong bias towards development of SW BPHs (*p* = 0.003, n = 100). (F) Increased expression of *Nlu-miR-34* (*p* = 6.04e^-5^), (G) significantly decrease of *NlInR1* (*p* = 0.008), (H) No effect on the expression of *NlInR2*. (I) JH application results in decreased abundance of *NlBr-C* transcripts (*p* = 0.022). The qPCR and wing rate data are presented as means ± SEM, three replicates. *p < 0.05, **p < 0.01, ***p < 0.001 (Student’s *t*-test).

Next, to further uncover the factors regulating *Nlu-miR-34* expression, we amplified the 5’UTR of *Nlu-miR-34* using rapid amplification of cDNA ends (RACE). The transcription starting site (TSS) was determined by mapping the 5’UTR sequence to the BPH genome (GenBank assembly accession: GCA_000757685.1), showing that the TSS of *Nlu-miR-34* is located at position 119,684 in scaffold0000007224(-). We searched for transcription binding sites (TFBS) nearby the TSS of *Nlu-miR-34* (-2,000 to +100) using PROMO [21] and Match [22]. Both programs identified a potential cis-response element (CRE) of Broad Complex (*Br-C*), suggesting that *NlBr-C* might involve in regulating *Nlu-miR-*34 (**Fig 6A**). To confirm this, we knocked down *NlBr-C* by injecting ds*NlBr-C* at the third instar nymphs in SW strain (**Fig 6B,** Student’s *t*-test, *p* = 3.21e^-5^, three replicates), resulted in the increase of *Nlu-miR-34* by 1.44-fold (**Fig 6C,** Student’s *t*-test, *p* = 0.011, three replicates). Because *Br-C* is an ecdysone inducible transcription factor [23], we then used 20-hydroxyecdysone (20E) to treat the third instar nymphs in SW strain. Topical application of 20E decreased *Nlu-miR-34* significantly (**Fig 6D**, Student’s *t*-test, *p* = 0.0001, three replicates). However, similar as the effect of using antagomir-34 alone without knocking down *NlInR2*, 20E application did not change the proportion of wing morphs.

Since JH and 20E show antagonistic actions through *Br-C* [24] and JH was known to regulate wing polyphenism in BPH [13, 25], we hypothesize that JH might involve in regulating the expression of *Nlu-miR-34*. To test this mode, we used topical application of JH III analogue at the third instar of BPHs in LW strain. Acetone were used as the negative control. The results showed that increased JH titer led to a strong bias towards SW BPHs (increasing by 23.09 ± 6.16%) (**Fig 6E**, Student’s *t*-test, *p* = 0.003, n = 100, three replicates). After JH application, *Nlu-miR-34* was significantly increased by 1.91-fold while *NlInR1* was decreased by 39.7 ± 14.26%, but it didn’t change the *NlInR2* level (**Fig 6F–6H**, Student’s *t*-test, *p* = 6.04e^-5^ and 0.008, three replicates). Moreover, *NlBr-C* was also decreased by 25.18 ± 12.13% at 24h post JH treatment (**Fig 6I**, Student’s *t*-test, *p* = 0.022, three replicates). These results showed that the upregulation of *Nlu-miR-34* by JH and the downregulation of *Nlu-miR-34* by 20E are likely mediated by *NlBr-C* in a transcriptional regulation manner.

### *Nlu-miR-34*, JH and *NlInR1* form a positive autoregulatory loop to control wing polyphenism

**Fig 7:**
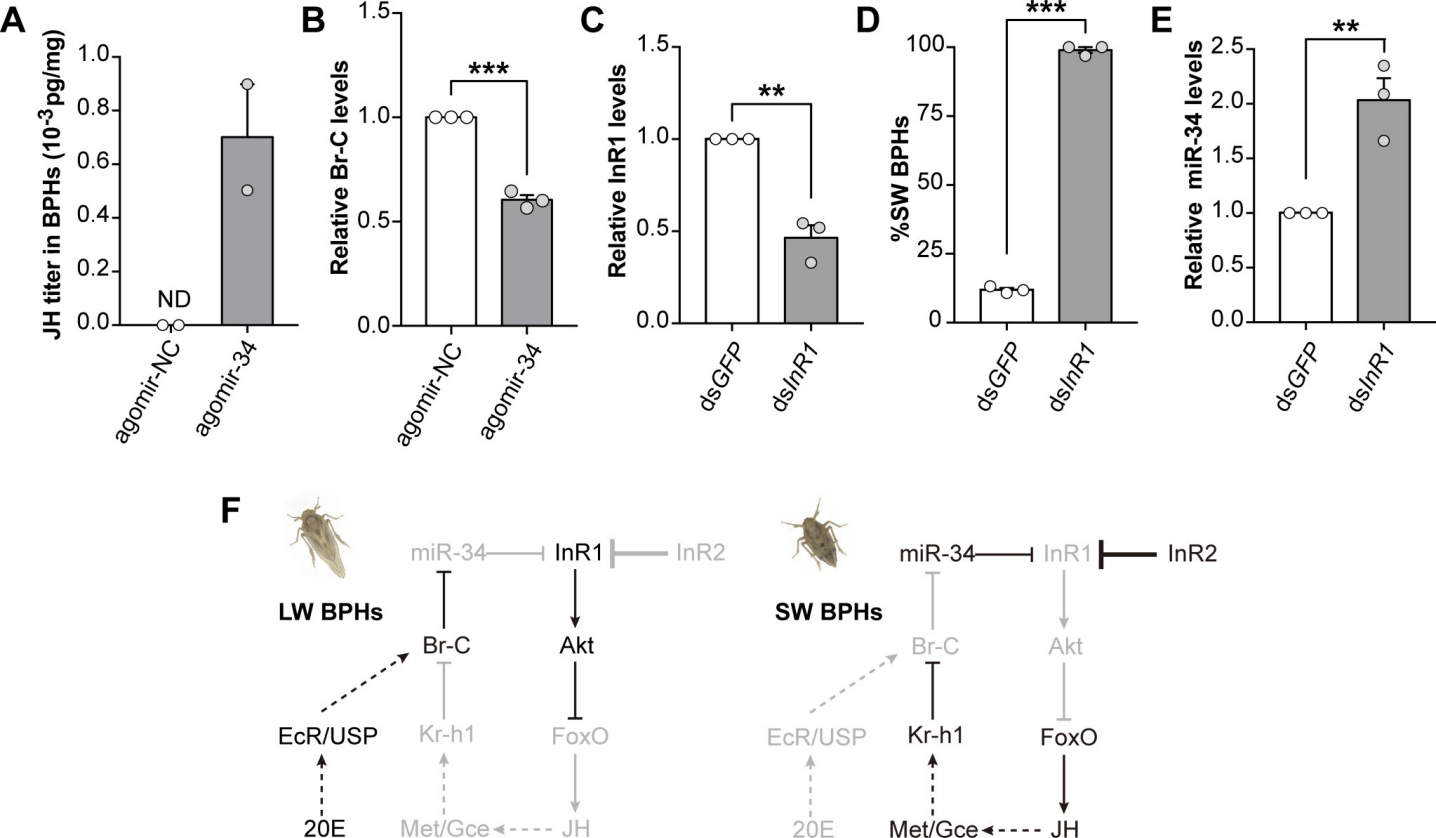
*Nlu-miR-34*, JH, 20E and *NlInR1* form a positive autoregulatory loop. (A) JH titers were increased significantly in BPHs after treated with agomir-34 in LW strain. ND: not detected. More than 300 insects were used for each experiment. (B) Injection of agomir-34 in LW strain resulted in decreased abundance of *NlBr-C* transcripts (*p* = 6.72e^-5^). (C) Knockdown of *NlInR1* significantly decreased the expression of *NlInR1* (*p* = 0.001). (D) Knocking down *NlInR1* in LW strain induced a strong bias towards SW BPHs (*p* = 2.51e^-7^, n = 100). (E) Higher transcriptional level of *Nlu-miR-34* in the knockdown SW BPHs (*p* = 0.006). The qPCR and wing rate data are presented as means ± SEM, three replicates. *p < 0.05, **p < 0.01, *** *p* < 0.001 (Student’s *t*-test). (F) Schematic model of the interplay between the IIS pathway, JH, 20E and miRNA during wing determination in BPHs. In LW BPHs, low transcriptional level of *Nlu-miR-34* fails to inhibit the expression of *NlInR1*, which activates *NlAkt*, inactivates *NlFoxO*, and reduces JH titer activates the activity of *NlBr-C*, which will maintain the low transcriptional level of *Nlu-miR-34*. In SW BPHs, high transcriptional level of *Nlu-miR-34* inhibits the expression of *NlInR1*, fails to inactivate *NlFoxO* by *NlAkt*, stimulating to a high JH titer and repression of *NlBr-C*, which will maintain the high transcriptional level of *Nlu-miR-34*. The components that are less active or inactive are shown in grey.

We previously showed that JH activates *Nlu-miR-*34. Interestingly, we found JH titer was significantly increased by the overexpression of *Nlu-miR-34* (**Fig 7A,** n>300), suggesting that JH and *Nlu-miR-34* form a positive autoregulatory loop. In addition, *NlBr-C* was significantly decreased by 39.60 ± 3.99% when overexpressing *Nlu-miR-34* (**Fig 7B**, Student’s *t*-test, *p* = 6.72e^-5^, three replicates), suggesting the positive feedback loop between JH and *Nlu-miR-34* might be mediated by *Br-C*.

To test whether *NlInR1* also involve in this autoregulatory loop, we next knocked down *NlInR1* by injecting ds*NlInR1* in the third instar nymphs in LW strain (**Fig 7C**). The results showed that knocking down *NlInR1* increased *Nlu-miR-34* by 2.03-fold (**Fig 7E**, Student’s *t*-test, *p* = 0.006, three replicates) and led to a strong bias towards SW morphs (**Fig 7D**, Student’s *t*-test, *p* = 2.51e^-7^, n = 100, three replicates). Taking together, these results suggested that *miR-*34, JH, 20E and IIS pathway form a positive autoregulatory loop to control wing polyphenism in BPHs (**Fig 7F**).

## Discussion

Many animals with the same genetic background exhibit polyphenism to adapt to different environment conditions [1]. It has been showed that insulin-like peptides (ILP), the IIS pathway, and JH involve in the regulation of polyphenism in various organisms [26–29]. However, it remains elusive how these regulators interact with each other. Here, we showed that *Nlu-miR-34* is a mediator between the crosstalk between IIS and hormonal signals by forming a positive feedback loop. miR-34 is a conserved miRNA that plays an important regulatory role in a wide range of organisms. It seems the positive feedback loop mediated by miR-34 might be a conserved process because *miR-34* is also regulated by IIS via a negative feedback loop between *miR-34* and *DAF-16* (the sole ortholog of *FoxO* in nematodes) in *C*. *elegans* [16]. We propose a regulation mode of JH -miRNA-IIS feedback loop in BPH (**Fig 7F**). Through this positive autoregulatory loop, minor changes in environment conditions, above a certain threshold, would be amplified significantly *in vivo* inducing a phenotype shift. Two pathways involved in miRNA or InR regulation are redundant or at least partially redundant. It seems that InR regulation is more sensitive to nutritive environment whereas miRNA regulation is likely to be hormonally regulated. This discovery extends our understanding of the interplay between nutrition status-based signals and hormone signals in modulating phenotypic plasticity.

Polyphenism is a phenomenon in which a single genotype can arise into two or more discrete phenotypes in response to environmental stimuli; these phenotypes do not segregate in F1 generations produced from parents with distinct phenotypes, suggesting that polyphenotypic changes are not determined by only one or two genes [30]. However, knocking down *NlInR1* induces formation of 100% SW BPHs [9], suggesting that the effect of insulin or hormone regulation is normally determinative in most cases. Therefore, other regulators should participate in regulation; and these regulators should be either sensitive to environmental cues or dose dependent. Here, we showed that *Nlu-miR-34* mediates the cross talk between JH and IIS, and miRNAs are normally expressed at moderate levels [31]. In contrast to other regulatory molecules, miRNAs are typically very fine-tuned, specifically binding to and regulating their targets, acting as buffers to ensure developmental robustness [32]. These features enable them to act as ideal regulators of phenotype polyphenism, responding to varied environment cues. To the best of our knowledge, we presented the first evidence that miRNAs regulate developmental plasticity by modulating the cross talk between the JH and IIS pathways. It should be noted that the experiments of overexpression of *Nlu-miR-*34 were repeated independently in three labs in Nanjing, Wuhan and Hangzhou, ensuring the reliability of miRNA modulation of wing polyphenism in BPH (**S4 Fig**).

JH was reported to regulate wing polyphenism in BPH twenty years ago by Tojo and his colleagues but without further evidence [13, 25]. A recent report showed that knocking down juvenile hormone epoxide hydrolase (*Jheh*) enhanced short wing formation [14]. However, Zhang and his colleagues argued that lack of direct evidence of JH regulation of wing polyphenism in BPH [8, 15]. Here we present evidence that JH participates in the regulation of wing polyphenism in BPH by upregulating *Nlu-miR-34* expression. JH has been reported to regulate miRNA expression during larvae development in *D*. *melanogaster* [18]. JH can redirect the development of LW to SW/wingless morphs in BPHs [13, 25], aphids [33], and crickets [34], but the relationship between JH and wing polyphenism is inverse with that in ants [35–37]. It is interesting to notice that both winged ants and SW BPHs have higher fecundity than other morphs, inferring JH regulation of wing polyphenism varies between different insects and JH might be involved in reproduction regulation [38], which requires further investigations.

Though JH has been well understood in regulating phenotype polyphenism [1, 28], the role of 20E is less understood in this field. We showed that *miR-34* is activated by JH but is suppressed by 20E in planthopper. A CRE of *Br-C* was found in the promoter region of *Nlu-miR-34*. It has been reported that downregulation of *miR-34* requires *Br-C* in *Drosophila* [18]. Since we did not find any JH response element (JHRE) in the promoter region of *Nlu-miR-34*, JH might regulate *Nlu-miR-34* in an indirectly manner. JH has been reported to show antagonistic interaction with 20E through *Br-C* [24], it is likely that JH may activate the expression of *Nlu-miR-34* through a cross talk between JH and 20E mediated by *Br-C*. However, topic application of 20E did not change the proportion of wing morphs. These results open a new question whether 20E regulates wing polyphenism in BPHs, and if so, in which way the 20E may regulate wing polyphenism.

In summary, we showed, for the first time, a comprehensive regulation mechanism of wing polyphenism in BPH by integrating almost all known regulators (miRNA, IIS, JH, and 20E) into a positive autoregulatory feedback loop (**Fig 7F**). We also presented evidence that 20E might also involve in regulating wing polyphenism by cross talking with JH. These discoveries extend our understanding of the mechanism regulating insect polyphenism, and shed new lights on how environmental cues determine which alternative phenotypes are produced by a given genotype.

## Material and methods

### Insects

Wild BPH populations were obtained from rice fields in Wuhan, Hubei Province, China, and raised on a BPH-susceptible rice variety Taichuang Native 1 (TN1) in the greenhouse. SW and LW strains were selected to propagate for more than 50 generations [14]. The percentage of SW morphs in the generated SW strain was more than 80%, while LW morphs represented approximately 80% of the LW strain. Each generation was purified in the adult stage using a previously described method [18]. BPHs were raised in growth chambers at 26°C (± 2°C) under a photoperiod of 16:8h (light: dark) with the relative humidity of 75% (± 5%).

### Prediction of miRNA targets

We obtained BPH miRNAs sequences that have been previously reported [39, 40]. The 3’UTR sequences of *NlInR1* (KF974333) and *NlInR2* (KF974334) were downloaded from NCBI (http://www.ncbi.nlm.nih.gov/). We used five algorithms, including miRanda [41], TargetScan [42], microTar [43], PITA [44], RNAhybrid [45], to predict miRNAs that can target *NlInR1* or *NlInR2*. Default parameters were used for all algorithms.

### microRNAs targeting *NlInR1*

To investigate whether miRNA may play a role in regulating wing polyphenism, we carried out a bioinformatics analysis to identify miRNAs targeting at *NlInR1* or *NlInR2*. The results showed that three miRNAs target on *NlInR1* and four miRNAs target on *NlInR2* (**S2 Table**). Then, we used luciferase reporter assays to confirm the interaction between miRNA and their targets, and the results confirmed the interactions between *NlInRs* and three miRNAs, *Nlu-miR-34*, *Nlu-miR-989b* and *Nlu-miR-989c*. We overexpressed these three miRNAs separately to test their effects on wing polyphenism in BPH, showing that only *Nlu-miR-34* can significantly change the ratio of wing morphs (**Fig 3A** and **S5 Fig**). So, we focused on *Nlu-miR-34* in further studies.

### Multiple sequences alignment of miR-34 sequences

miR-34-5p sequences from several insects and nematode *C*. *elegans* were downloaded from miRBase and submitted to ESPript 3.0 (http://espript.ibcp.fr/ESPript/cgi-bin/ESPript.cgi) for multiple sequence alignment and visualization.

### *In vitro* luciferase assays

The 3’UTRs of *NlInR1* and *NlInR2* were amplified and introduced into the pMIR-REPORT vector (Obio, China) downstream of the firefly luciferase gene. The pRL-CMV vector (Promega, USA), which contains the *Renilla* luciferase gene, was used as a control luciferase reporter vector. The predicted binding site (5’-CACTAGTGACCGCGTAGTTGCCTGCCG-3’) was completely removed for mutant 1, and another binding site (5’-TGACGTTGCCGCCGCCACTGCCG-3’) was removed for generation of mutant 2. HEK293T cells (Obio, China) were cultured at 37°C in 5% CO_2_ in DMEM (Gibco, USA) media supplemented with 10% FBS (Hyclone) for 24 h in 96-well culture plates. Cells were transfected with pMIR-REPORT (0.2 μg), pRL-CMV (0.01μg), 0.25 μl of 100 nM miRNA mimics (RiboBio, China), and 0.25 μl Lipofectamine 2000 reagent (Invitrogen, USA) according to the manufacturer’s instructions. The activity of the two luciferase enzymes was measured 48 h after transfection following the manufacturer’s recommendations (Dual-Luciferase Reporter Assay System, Promega, USA) with Infinite M1000 (Tecan, Switzerland). Three replicates were performed for each experiment. Results are expressed as the means of the ratio of firefly luciferase activity/Renilla luciferase activity. Controls were set to one, and the two-tailed *t-*test was used for statistical analysis.

### RNA-binding protein immunoprecipitation Assay (RIP)

A Magna RIP Kit (Millipore, Germany) was used to perform the RIP assay according to the manufacturer’s instructions. Approximately 50 wing buds of the fourth instar nymphs were collected and crushed using a homogenizer in ice-cold RIP lysis buffer. Magnetic beads were incubated with 5 μg of RIPAb+ Ago-1 antibody (Millipore, Germany) or normal mouse IgG (Millipore, Germany). The homogenates in the RIP lysates were centrifuged and the Ago 1-bound mRNAs in supernatants were pulled down by magnetic bead–antibody complex at 4°C overnight. The immunoprecipitated RNAs were released by digestion with protease K. Finally, the RNAs were purified by which methods and used for cDNA synthesis. The PrimeScript 1^st^ Strand cDNA Synthesis Kit (Takara, Japan) was used and the abundance of *InR1* was quantified by qPCR.

### Fluorescence *in situ* hybridization (FISH)

An antisense nucleic acid detection probe (5’-ACAACCAGCUAACCACACUGCCA -3’) designed to detect *Nlu-miR-34* was labeled with Cy3. The probe for detecting *NlInR1* (5’- GAACAGCCAGGACAGGCCGAAUCCUCCAUG-3') was labeled with FAM. The random shuffled probe (5’-UUCUCCGAACGUGUCACGUU-3’) and the probe (5’-UUGUACUACAAAAGUACUG-3’) were used as miRNA and mRNA negative controls, respectively. The wing buds were dissected from the third or fourth instar stage nymphs injected with agomir or antagomir (RiboBio, China). For fluorescence *in situ* hybridization, wing buds were treated for 24 h, fixed in 4% paraformaldehyde for 2 hours, and then incubated with the miRNA probes at 37°C for 24 h. The samples were washed in PBS containing Triton X-100 (5% v/v) and then incubated with the DAPI (RiboBio, China) at room temperature for 30 min. Signals were analyzed and images were recorded using a Zeiss LSM 780 confocal microscope (Carl Zeiss SAS, Germany). Figures were prepared using Zeiss LSM ZEN 2010 software (Carl Zeiss SAS, Germany).

### 5’UTR amplification and promoter analysis of *Nlu-miR-34*

Total RNA was isolated from ten fourth to fifth instar nymphs of SW BPHs using the TRIzol regent (Invitrogen, USA). The SMARTer RACE cDNA amplification kit (Clontech, USA) was used to obtain the 5’UTR of *Nlu-miR-34*. 5’RACE nested-PCR was performed using *Ex-Taq* (Takara, Japan) according to the manufacturer’s instructions. The amplified products were separated by agarose gel electrophoresis and purified using a Gel Extraction Kit (Takara, Japan). The purified DNA was ligated into the pMD19-T vector (Takara, Japan), and sent to BGI-Shenzhen for sequencing.

The transcript starting site (TSS) of *miR-34* was confirmed by alignment to genomic scaffold sequences (GenBank assembly accession: GCA_000757685.1). Any unknowns “N” in the genomic sequences were confirmed by PCR validation. A 2100 bp flanking sequence (-2000 to +100) upstream of the TSS was used as the promoter region for analysis by Promoter Scan web server (http://www.cbs.dtu.dk/services/Promoter/). A putative TATA-box was identified at position -1707. To identify putative transcription factor binding sites (TFBS) in the promoter region, two algorithms, including PROMO 3.0 [18] and MATACH 1.0 [19] based on TRANSFAC database (http://gene-regulation.com/pub/databases.html), were computationally analyzed using default parameters. A *Br-C Z4* biding site (TTTTGTTTAAATT) at position -699 was predicted by both programs. The *Nlu-miR-34* sequence, including the intact 5’UTR, was deposited in GenBank (MG894367).

### Injection of agomir-34 and antagomir-34

BPH nymphs were collected for microinjection on the first day of the third instar. 15 nl of agomir-34 (40 ng, 15 nl) or antagomir-34 (40 ng, 15 nl) were microinjected into the conjunctive of each nymph between the prothorax and mesothorax using a Nanoliter 2000 injector (WPI, USA). 150 nymphs were used for each experiment. Ten nymphs were transferred into glass tubes 24 h post injection and placed in a growth chamber. Agomir or antagomir of random shuffled sequences was injected with equivalent volume as negative control (RiboBio, China). All experiments were performed in triplicate.

### RNA interference (RNAi)

In order to knock down *NlInR1* and *NlBr-C* expression, dsRNA was synthesized using the T7 RiboMAXTM Express RNAi System (Promega, USA) following the manufacturer’s instructions. Each nymph, at the first day of the third instar, was injected with dsRNA (250 ng, 15 nl) using a Nanoliter 2000 injector (WPI). Equivalent volumes of ds*GFP* were injected as negative controls. 100 nymphs were used for each treatment, and all experiments were carried out in triplicate.

### Co-injection of ds*NlInR2* and antagomir

BPH nymphs were collected for microinjection on the first day of the third instar. The ds*NlInR2*, the concentration of which were divided into three groups, 0.42 ng, 0.84 ng and 3.38 ng, was injected into nymphs respectively with 40 ng antagomir-34. Either 40 ng or 60 ng antagomir-34 was injected into nymphs with 0.84 ng ds*NlInR2*. The volumes of mixtures were 15 nl. All of microinjections were done using a Nanoliter 2000 injector (WPI, USA). Approximately 200 nymphs were used for each experiment. Control nymphs were injected with equivalent volumes of the mixtures of ds*NlInR2* and antagomir-NC. All experiments were performed in triplicate.

### Topical application of JH

Juvenile hormone III (JH III, 96%) was purchased from Toronto Research Chemicals (Sigma, USA). 200 ng of JH III was dissolved in acetone and dropped 40 nl onto the mesonotum of the third instar BPH nymphs using a Nanoliter 2000 injector (WPI, USA), after anaesthetizing with carbon dioxide [14]. Equivalent volumes of acetone were used as negative controls. The rearing condition and methods of observation were same as the method of RNAi and injection of agomir-34 and antagomir-34. 100 nymphs were used for each experiment, all experiments were performed in triplicate.

### Quantitative real-time PCR

Total RNA, enriched for miRNAs and mRNA, was extracted using the miRNeasy Mini kit (Qiagen, Germany) and TransZol Up Plus RNA kit (TransGen, China) from whole insect bodies (n = 30), respectively. The miScript Ⅱ RT kit (QIAGEN) and the TransScript One-Step gDNA Removal and cDNA Synthesis SuperMix kit (TransGen) were used to prepare cDNA. Quantitative real-time PCR (qPCR) reactions were conducted with an ABI Prism 7300 (Applied Biosystems, USA) using miScript SYBR Green (Qiagen, Germany) and SYBR® Premix Ex Taq II RR820A (Takara, Japan), according to the manufacturer’s instructions. The data was analyzed using the 2^-△△Ct^ method. Actin1 (GenBank accession No. EU179846.1) was used as the endogenous control. This gene has been studied to show it is suitable to be used as a reference gene [46]. All treatments were carried out in triplicate. Independent reactions were performed in quadruplicate for each RNA sample, and the signal intensity of the target gene is presented as the average value. The qPCR primers used in this study are listed in **S3 Table**.

### Juvenile hormone estimation by LC-MS/MS

About 300 BPHs were collected and pooled together for analysis. Extraction of JH III was performed according to a previously described method with some modifications [47, 48]. JH III dilutions of 20, 10, 1, 0.5, 0.25, 0.125, and 0.0625 ng/ml were prepared in 70% methanol and used for calculation of the standard curve. The LC–MS/MS analyses were performed using an Agilent 6460 triple quadrupole mass spectrometer (Agilent, USA) equipped with an electrospray ionization (ESI) source, operated in the positive ion multiple-reaction monitoring (MRM) mode. Agilent Mass Hunter Workstation software was used for system operation, data acquisition, and data analysis. Chromatographic separation was carried out using a Zorbax SB-Aq column (100 mm × 2.1 mm, 3.5 μm) (Agilent, USA). JH III was separated using a binary gradient. Briefly, mobile phase A consisted of 0.1% formic acid in water and mobile phase B was comprised of 0.1% formic acid in acetonitrile. The gradient program was as follows: 0–5 min, 60%–85% of B; 5–8 min, 85%–95% of B. The flow rate of the mobile phase and the column temperature were set at 0.3 mL/min and 30°C, respectively, and the injection volume was 5 μl. The retention time of JH III was 3.82 min and the total run time was 10 min. Mass spectrometric detection was completed by use of an electrospray ionization (ESI) source in positive ion multiple-reaction monitoring (MRM) mode using the following parameters: precursor (m/z), 267; ion product (m/z), 235.2; fragmentor voltage (V), 80; collision Energy (eV), 1.

### Statistical analysis

SPSS 17.0 software (IBM SPSS Inc. USA) was used for statistical analysis. The differences between treatments were compared using the Student’s *t*-test. The effects of antagomir on distribution of wing types in BPHs were analyzed using the Chi-square test. *p* < 0.05 was considered statistically significant. All results are expressed as means ± SEM, three replicates.

## Supporting information

S1 Fig *Nlu-miR-34* is co-localized with *NlInR1* in the cells of wing buds of the third instar SW strain nymphs. Green (*NlInR1*) and red (*Nlu-miR-34*) signals overlap to show yellow signals, suggesting *Nlu-miR-34* interact directly with *NlInR1* in the cells of wing buds. Control 1, *Nlu-miR-34* antisense and scrambled mRNA probe; Control 2, scrambled miRNA and *NlInR1* antisense probe; Control 3, scrambled miRNA and scrambled mRNA probe. Scale bars: 100 μm.
(TIF)

S2 Fig Expression of *NlInR1* in different development stages in BPHs. The data are presented as means ± SEM, three replicates. *p < 0.05 (Students’ *t*-test).
(TIFF)

S3 Fig Expression of *NlInR2* in different development stages in BPHs. The data are presented as means ± SEM, three replicates. *p < 0.05, **p < 0.01 (Students’ *t*-test).
(TIFF)

S4 Fig Overexpression of *Nlu-miR-*34 were repeated independently in three labs in Hangzhou (p = 0.0002), Nanjing (p = 0.009) and Wuhan. The data are presented as means ± SEM, three replicates. **p < 0.01, ***p < 0.001 (Students’ *t*-test).
(TIFF)

S5 Fig Dual luciferase reporter assays and overexpression of miRNAs which target *NlInR1* and *NlInR2*. (A) Dual luciferase reporter assays confirmed the interactions between *Nlu-miR-34* and *NlInR1 in vitro* (*p* = 2.35e^-13^). (B, C) Dual luciferase reporter assays confirmed the interactions between *Nlu-miR-989b* and *NlInR2* (*p* = 1.43e^-12^), and the interactions between *Nlu-miR-989c* and *NlInR2 in vitro* (*p* = 9.48e^-13^). Data are means ± SEM, six replicates. (D, E) Overexpression of *Nlu-miR-989b* and *Nlu-miR-989c* in SW-strain. Wing rate data are presented as means ± SEM, three replicates. ***p < 0.001 (Student’s *t*-test).
(TIFF)

S1 Table Predicted target genes of *Nlu-miR-34* in BPH genome. (DOCX)

S2 Table The miRNAs predicting to target *InRs*. (DOCX)

S3 Table Primers used in this study. (DOCX)
