# Supplementary figures and images for: miR-34 modulates wing polyphenism in planthopper
Source: PLoS Genet. 2019 Jun 26;15(6):e1008235. doi: 10.1371/journal.pgen.1008235 (PMC6615638; doi:10.1371/journal.pgen.1008235)

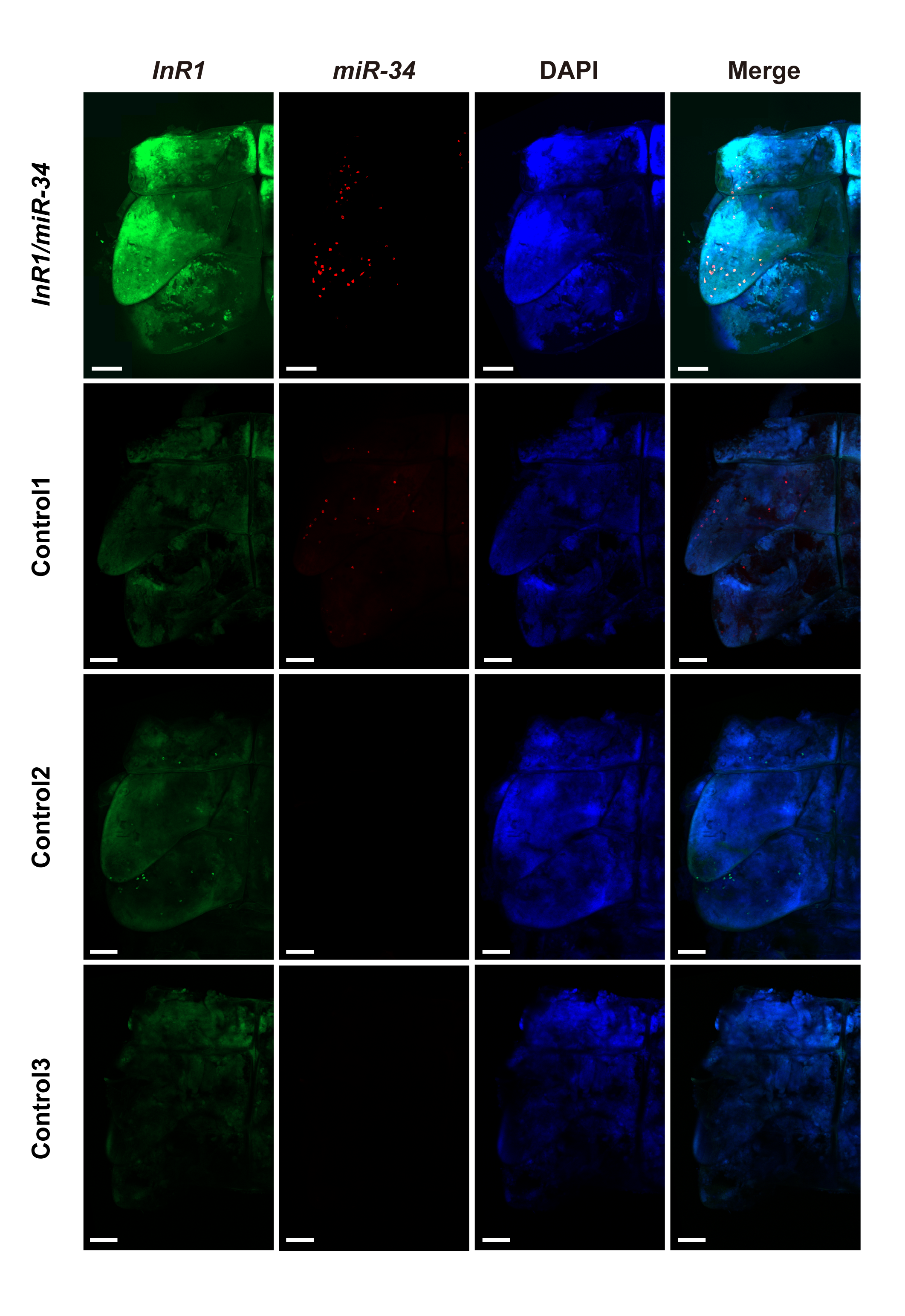

Supplement: S1 Fig — Green (NlInR1) and red (Nlu-miR-34) signals overlap to show yellow signals, suggesting Nlu-miR-34 interact directly with NlInR1 in the cells of wing buds. Control 1, Nlu-miR-34 antisense and scrambled mRNA probe; Control 2, scrambled miRNA and NlInR1 antisense probe; Control 3, scrambled miRNA and scrambled mRNA probe. Scale bars: 100 μm. (TIF) [file pgen.1008235.s001.tif]

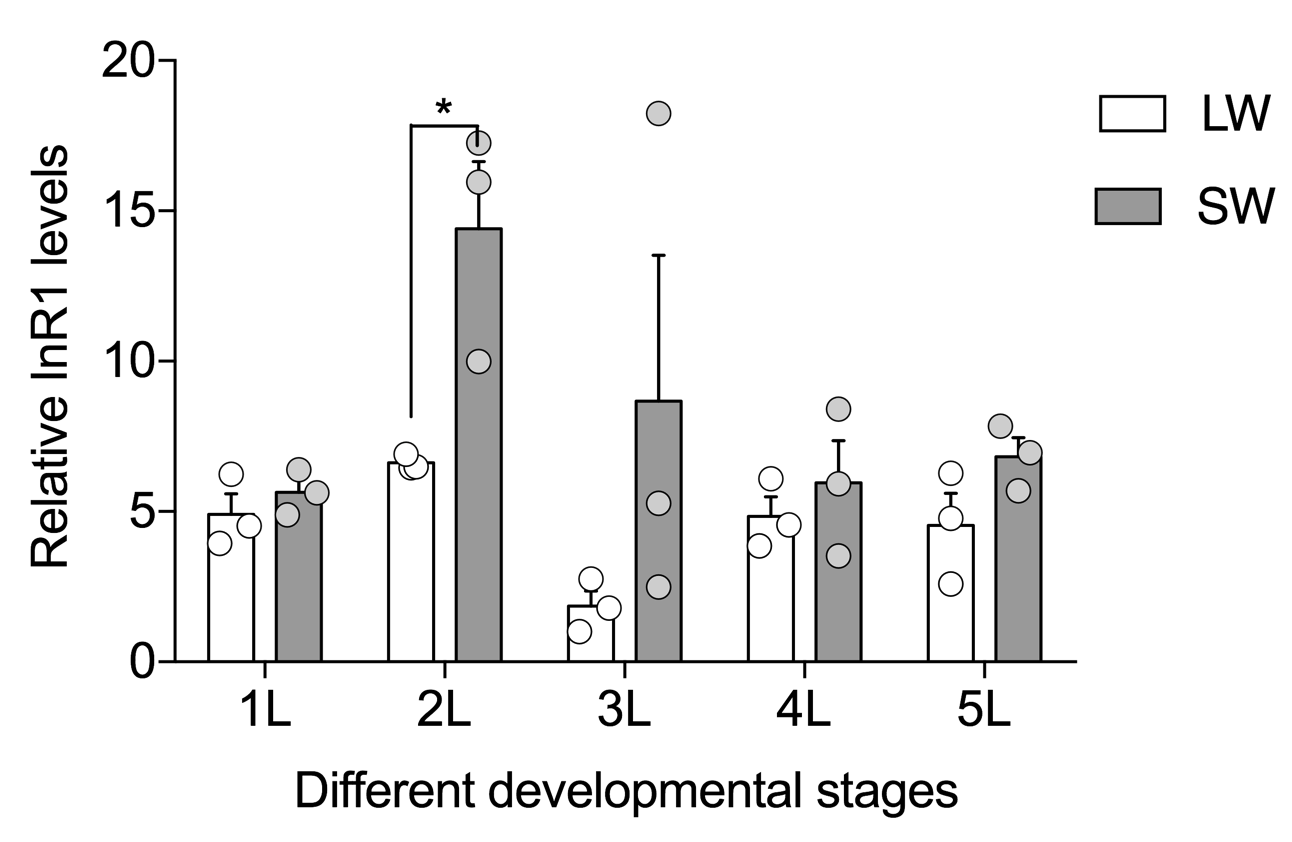

Supplement: S2 Fig — The data are presented as means ± SEM, three replicates. *p < 0.05 (Students’ t-test). (TIFF) [file pgen.1008235.s002.tiff]

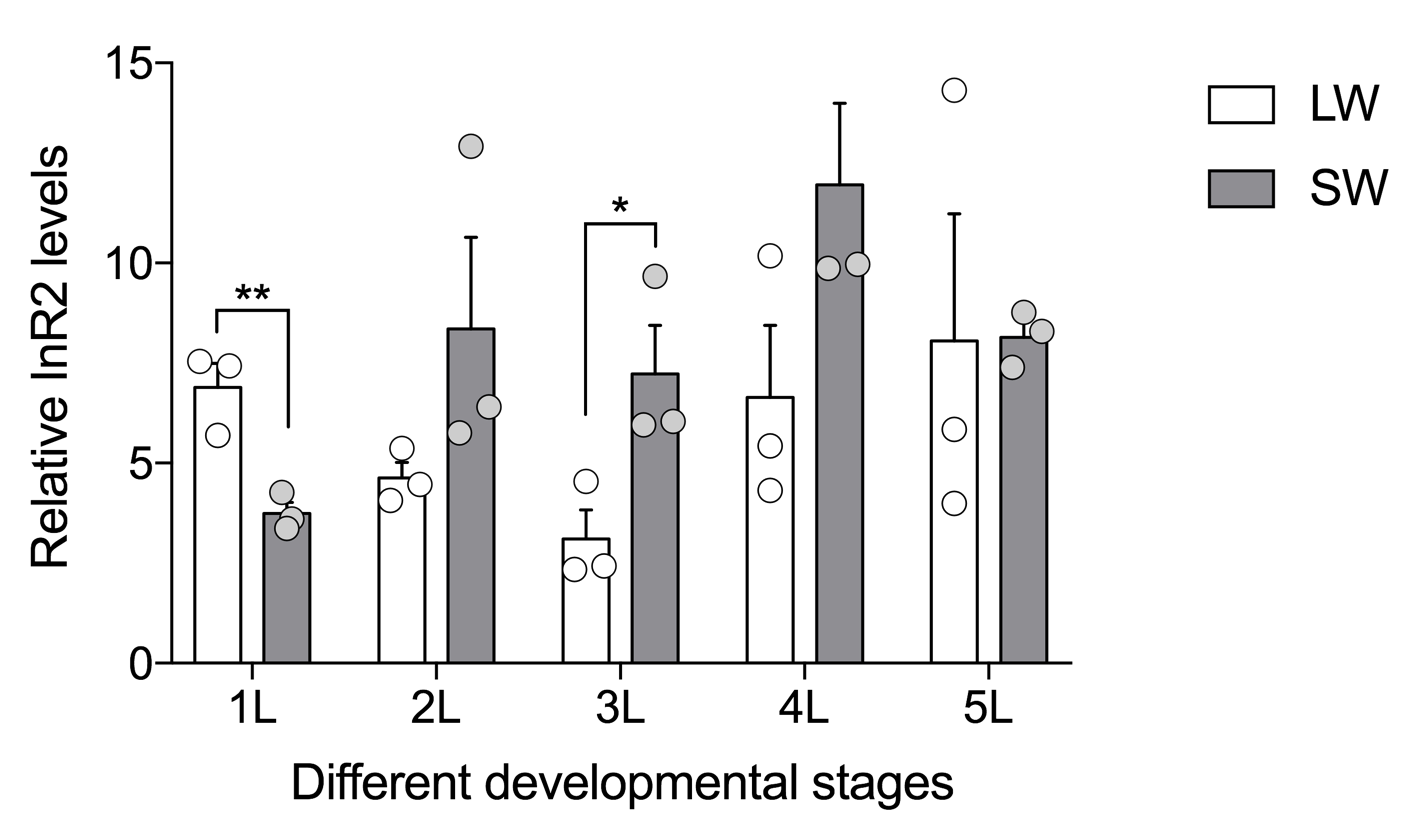

Supplement: S3 Fig — The data are presented as means ± SEM, three replicates. *p < 0.05, **p < 0.01 (Students’ t-test). (TIFF) [file pgen.1008235.s003.tiff]

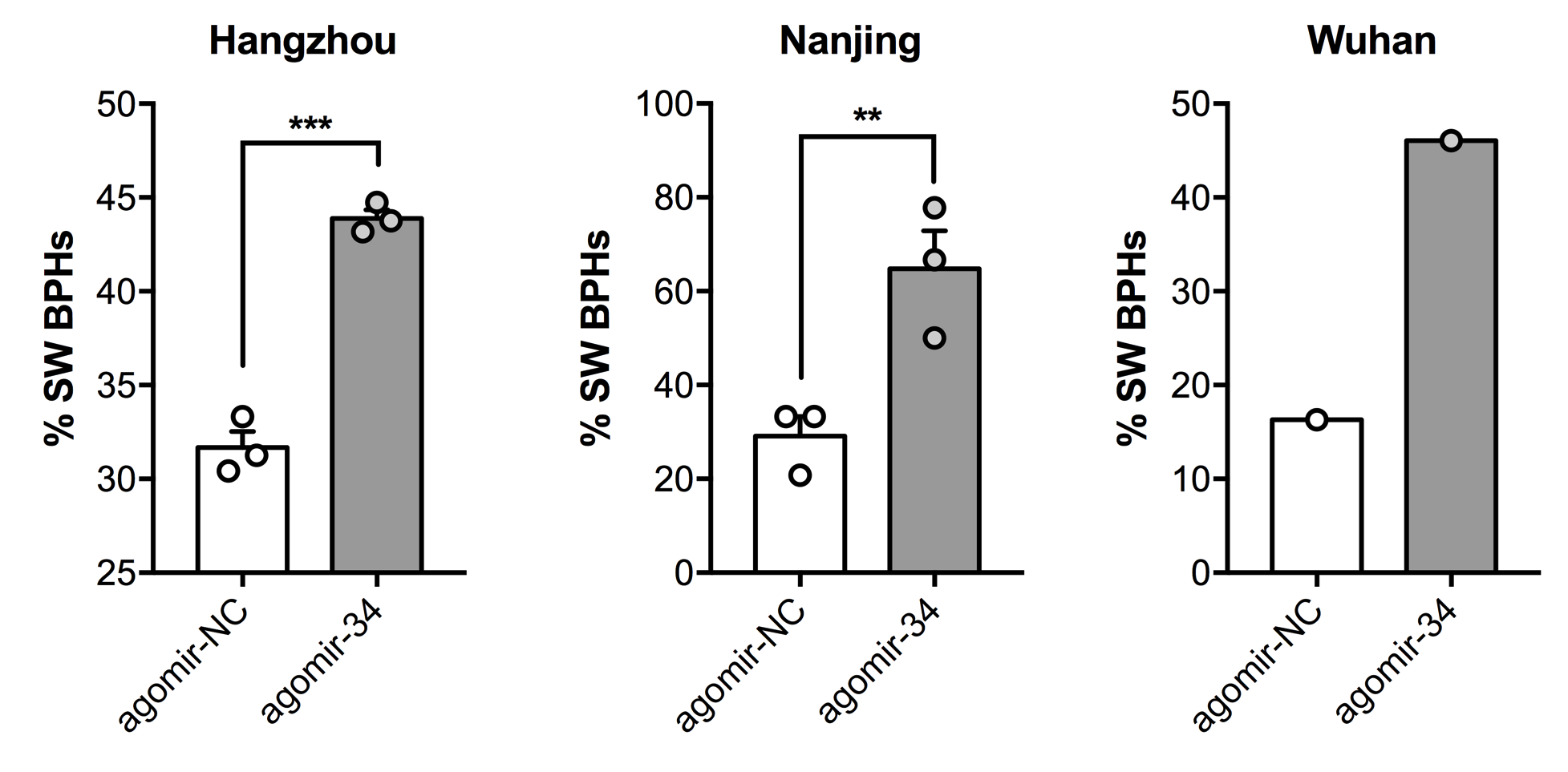

Supplement: S4 Fig — The data are presented as means ± SEM, three replicates. **p < 0.01, ***p < 0.001 (Students’ t-test). (TIFF) [file pgen.1008235.s004.tiff]

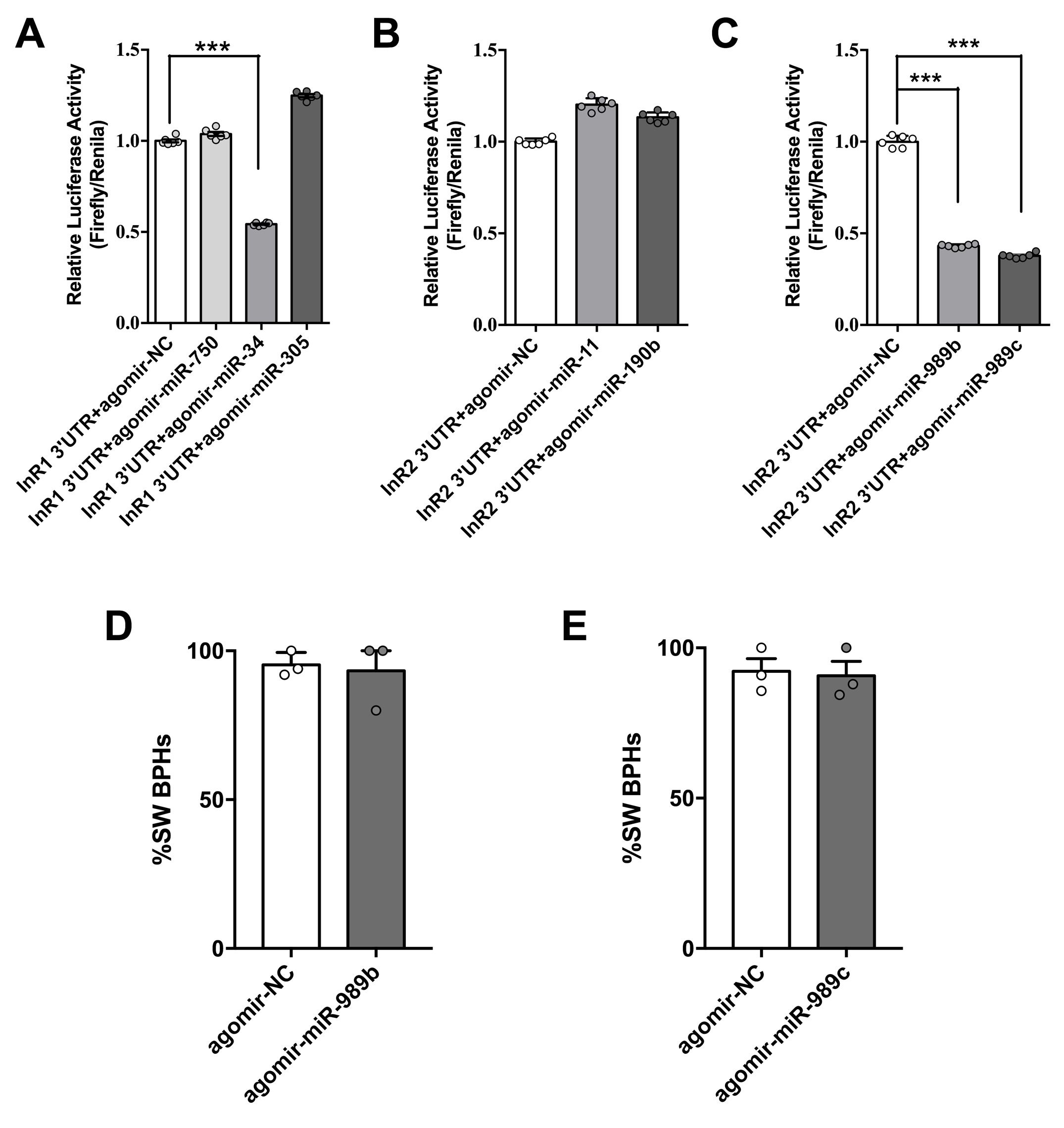

Supplement: S5 Fig — (A) Dual luciferase reporter assays confirmed the interactions between Nlu-miR-34 and NlInR1 in vitro (p = 2.35e-13). (B, C) Dual luciferase reporter assays confirmed the interactions between Nlu-miR-989b and NlInR2 (p = 1.43e-12), and the interactions between Nlu-miR-989c and NlInR2 in vitro (p = 9.48e-13). Data are means ± SEM, six replicates. (D, E) Overexpression of Nlu-miR-989b and Nlu-miR-989c in SW-strain. Wing rate data are presented as means ± SEM, three replicates. ***p < 0.001 (Student’s t-test). (TIFF) [file pgen.1008235.s005.tiff]
