# Supplementary material for: miR-34 modulates wing polyphenism in planthopper
Source: PLoS Genet. 2019 Jun 26;15(6):e1008235. doi: 10.1371/journal.pgen.1008235 (PMC6615638; doi:10.1371/journal.pgen.1008235)
Supplement: S1 Table — (DOCX) [file pgen.1008235.s006.docx]

**S1 Table.** Predicted target genes of *Nlu-miR-34* in BPH genome.

| Target gene ID | Annotation |
| --- | --- |
| BPHOGS10024100-TA | Insulin receptor 1 |
| BPHOGS10016624-TA | AN1-type zinc finger protein 4 |
| BPHOGS10017748-TA | Centrosomal protein of 131 kDa |
| BPHOGS10015440-TA | Phthiocerol synthesis polyketide synthase type I PpsC |
| BPHOGS10035678-TA | ATP synthase lipid-binding protein, mitochondrial |
| BPHOGS10012043-TA | T-complex protein 1 subunit beta |
| BPHOGS10017815-TA | Glutathione peroxidase 3 |
| BPHOGS10019091-TA | Serine/threonine-protein kinase ATR |
| BPHOGS10014833-TA | Probable Na(+)/H(+) antiporter C3A11.09 |
| BPHOGS10025794-TA | Serine protease |
| BPHOGS10009469-TA | Putative exonuclease GOR |
| BPHOGS10011009-TA | ES1 protein homolog, mitochondrial |
| BPHOGS10021720-TA | ES1 protein homolog, mitochondrial |
| BPHOGS10015017-TA | RNA-binding protein 25 |
| BPHOGS10015016-TA | RNA-binding protein 25 |
| BPHOGS10033212-TA | Regulator of rDNA transcription protein 15 |
| BPHOGS10027255-TA | Putative uncharacterized protein ART3 |
| BPHOGS10000426-TA | Chitin binding Peritrophin-A domain |
| BPHOGS10021457-TA | hypothetical protein |
