# Supplementary material for: miR-34 modulates wing polyphenism in planthopper
Source: PLoS Genet. 2019 Jun 26;15(6):e1008235. doi: 10.1371/journal.pgen.1008235 (PMC6615638; doi:10.1371/journal.pgen.1008235)
Supplement: S2 Table — (DOCX) [file pgen.1008235.s007.docx]

**S2 Table.** The miRNAs predicting to target *InRs*.

| Target gene | miRNA | microTar | miRanda | PITA | RNAhybrid | TargetScan |
| --- | --- | --- | --- | --- | --- | --- |
| *InR1* | mir-750-5p | 🗸 | 🗸 | 🗸 | 🗸 | 🗸 |
|  | mir-34-5p | 🗸 | 🗸 | 🗸 | - | 🗸 |
|  | mir-305-5p | - | 🗸 | 🗸 | 🗸 | 🗸 |
| *InR2* | mir-11-5p | 🗸 | 🗸 | 🗸 | 🗸 | - |
|  | mir-190b-3p | 🗸 | 🗸 | 🗸 | - | 🗸 |
|  | mir-989b-5p | 🗸 | 🗸 | 🗸 | - | 🗸 |
|  | mir-989c-5p | 🗸 | 🗸 | 🗸 | - | 🗸 |
