# Supplementary material for: miR-34 modulates wing polyphenism in planthopper
Source: PLoS Genet. 2019 Jun 26;15(6):e1008235. doi: 10.1371/journal.pgen.1008235 (PMC6615638; doi:10.1371/journal.pgen.1008235)
Supplement: S3 Table — (DOCX) [file pgen.1008235.s008.docx]

**S3 Table.** Primers used in this study.

| Primer name | Sequence (5’-3’) | Length | Purpose |
| --- | --- | --- | --- |
| ds*InR1*-F | TAATACGACTCACTATAGGCGCTCTGGTTGTGCTTGATA | 437bp | *NlInR1* dsRNA synthesis |
| ds*InR1*-R | TAATACGACTCACTATAGGCGTTGTCTTTCTCCAACGGT |  |  |
| ds*InR2*-F | TAATACGACTCACTATAGGTCGCTATCAGCTGCTCAGAA | 451bp | *NlInR2* dsRNA synthesis |
| ds*InR2*-R | TAATACGACTCACTATAGGACGGAAATTTGTCTCCGTTG |  |  |
| ds*Akt*-F | TAATACGACTCACTATAGGTGCCATTCTACAACCGCGATC | 414bp | *NlAkt* dsRNA synthesis |
| ds*Akt*-R | TAATACGACTCACTATAGGAAGCCATGTCCTGGTAGCTG |  |  |
| ds*Foxo*-F | TAATACGACTCACTATAGGGAACTCGATCAGACACAACCT | 361bp | *NlFoxo* dsRNA synthesis |
| ds*Foxo*-R | TAATACGACTCACTATAGGGCGACGGCCGGAATCGGCGAC |  |  |
| ds*JHEH*-F | TAATACGACTCACTATAGGGCACGATTCAGCCTTCACCTA | 443bp | *NlJHEH*dsRNA synthesis |
| ds*JHEH*-R | TAATACGACTCACTATAGGCCCAGTCACCACCTTGGACAT |  |  |
| ds*GFP*-F | TAATACGACTCACTATAGGACGTAAACGGCCACAAGTTC | 495bp | *GFP* dsRNA synthesis |
| ds*GFP*-R | TAATACGACTCACTATAGGTGTTCTGCTGGTAGTGGTCG |  |  |
| 3’UTR-*InR1*-F | CCACTAGTGACCGCGTAGTTG | 1182bp | *NlInR1* 3’UTR synthesis |
| 3’UTR-*InR1*-R | GGTGGAGTGATTTTATTACAGCTC |  |  |
| Q-*actin*-F | CCAACCGTGAGAAGATGACC | 296bp | qRT-PCR for *Nlactin* |
| Q-*actin*-R | GATGTCACGCACGATTTCAC |  |  |
| Q-*InR1*-F | GAGTGCAACCCGGAGTATGT | 172bp | qRT-PCR for *NlInR1* |
| Q-*InR1*-R | TCTTGACGGCACACTTCTTG |  |  |
| Q-*InR2*-F | CTCTTGCCGAACAGCCTTAC | 152bp | qRT -PCR for *NlInR2* |
| Q-*InR2*-R | GGGTCGTTTAGTGGGTCTGA |  |  |
| Q-*JHEH*-F | CCACGAAGCCAGACACAATT | 190bp | qRT -PCR for *NlJHEH* |
| Q-*JHEH*-R | ATAGTTCCCGTCACCCAGTAG |  |  |
| 34-5-GSP | TGTGGGAAGTGGCCGTGTGTGGTAC | 845bp | 5’UTR amplification of miR-34 |
| 34-5-NGSP | CCAGCTAACCACACTGCCAACATGTCA | 818bp |  |
